# The digitization process and the evolution of Clinical Risk Management concept: The role of Clinical Engineering in the operational management of biomedical technologies

**DOI:** 10.3389/fpubh.2023.1121243

**Published:** 2023-02-02

**Authors:** Matteo Verga, Gian Luca Viganò, Martina Capuzzo, Claudia Duri, Lucia Maria Ignoti, Paola Picozzi, Veronica Cimolin

**Affiliations:** ^1^ASST Spedali Civili di Brescia - SC Ingegneria Clinica, Brescia, Italy; ^2^Department of Electronics, Information and Bioengineering, Politecnico di Milano, Milan, Italy; ^3^S. Giuseppe Hospital, Istituto Auxologico Italiano, IRCCS, Piancavallo, Italy

**Keywords:** digitization process, digital transformation, thematic evolution, clinical engineering, operational management, clinical risk management, biomedical technologies, management software

## Abstract

**Introduction:**

Digital transformation and technological innovation which have influenced several areas of social and productive life in recent years, are now also a tangible and concrete reality in the vast and strategic sector of public healthcare. The progressive introduction of digital technologies and their widespread diffusion in many segments of the population undoubtedly represent a driving force both for the evolution of care delivery methods and for the introduction of new organizational and management methods within clinical structures.

**Methods:**

The CS Clinical Engineering of the “Spedali Civili Hospital in Brescia” decided to design a path that would lead to the development of a software for the management of biomedical technologies within its competence inside the hospital. The ultimate aim of this path stems from the need of Clinical Engineering Department to have up-to-date, realistic, and systematic control of all biomedical technologies present in the company. “Spedali Civili Hospital in Brescia” is not just one of the most important corporate realities in the city, but it is also the largest hospital in Lombardy and one of the largest in Italy. System development has followed the well-established phases: requirement analysis phase, development phase, release phase and evaluating and updating phase.

**Results:**

Finally, cooperation between the various figures involved in the multidisciplinary working group led to the development of an innovative management software called “*SIC Brescia*”.

**Discussion:**

The contribution of the present paper is to illustrate the development of a complex implementation model for the digitization of processes, information relating to biomedical technologies and their management throughout the entire life cycle. The purpose of sharing this path is to highlight the methodologies followed for its realization, the results obtained and possible future developments. This may enable other realities in the healthcare context to undertake the same type of pathway inspired by an accomplished model. Furthermore, future implementation and data collection related to the proposed Key Performance Indicators, as well as the consequent development of new operational management models for biomedical technologies and maintenance processes will be possible. In this way, the Clinical Risk Management concept will also be able to evolve into a more controlled, safe, and efficient system for the patient and the user.

## 1. Introduction

### 1.1. Contest of reference

We live in an era in which we have witnessed and continue to witness birth, development and progressive spread of digital infrastructures and tools. The magnitude of this change makes it possible to assert that what we are facing today is a true digital revolution that influences and changes the paradigms in which we live, operate, and conduct our daily work (1). Digital transformation and technological innovation which have influenced several areas of social and productive life in recent years, are now also a tangible and concrete reality in the vast and strategic sector of public healthcare (2, 3). The progressive introduction of digital technologies and their widespread diffusion in many segments of the population undoubtedly represent a driving force both for the evolution of care delivery methods—which are increasingly precise and personalized—and for the introduction of new organizational and management methods within clinical structures (4, 5). Indeed, it can be asserted that the reformative thrust of digital health has a strong impact not only in the evolution of the delivery of clinical therapies in support of the patient, but it can also find significant applications in the context of the activities related to the management of biomedical technologies and the development of the models of what is nowadays defined as “Operational Management (OM)” (6, 7). So, a real transformation in a digital key consequently requires a not easy changing in the technological, structural, and organizational assets that its implementation imposes; thus, producing a new managerial structure that contemplates new aspects or new opportunities to deepen (8). Among the new aspects related to this renewed management, it is necessary and proper to emphasize how a biomedical technology today can no longer be seen as a stand-alone and independent element—even where it was—but rather, it becomes an element active part of a larger system. In this increasingly articulated system, within which it is placed, its role becomes fundamental to the functioning of the embedded process. A context that wants to evolve its organization and that aims to improve quality and efficiency of health services, must therefore be able to obtain structured and multi-parametric information; not easy way because this aspiration implies in having to face new challenges (9). The most important one is represented by the establishment of new information flows that require a logic of compatibility, interoperability and strong integration between technologies and the rest of—local, regional and national—information systems. This aggregate overview suggests the need for increasingly structured data collection and its subsequent processing for construction of dashboards with summary data and Key Performance Indicators (KPIs) (10, 11). This approach allows the optimization of resources according to economic (management costs) and organizational (resources) requirements, quality, regulatory and safety reference standards (12, 13). Against this backdrop, the challenge for today's Clinical Engineering Departments (CED) is to embark on a digitization path of technologies and processes (14–16). The latter is represented by defining a new way of mapping and managing medical equipment and medical devices distributed at a territorial level, even more so at home (in the logic of proximity medicine), throughout their entire life cycle, considering all the possible needs that can be encountered, ranging from simple periodic maintenance to extraordinary maintenance to technological and/or software upgrades, to the renewal of any necessary consumables. Last but not least, the safe use of medical device according to its specific destination. Thus, in this articulated scenario, a new range of action and supervision is established for the Clinical Engineer, a professional figure which is able to move from a hospital-centric logic to a digitized territorial logic. The role of CED, within healthcare facilities, is to participate in health care and to ensure the safe, appropriate, and economical use of biomedical technology. They are therefore specialized in optimizing the management of healthcare equipment for hospital use. One of their main tasks is to balance the need for optimization of healthcare expenditure and the quality of service rendered to the end-patient. For this reason, they carry out very cross-disciplinary studies, touching on the traditional worlds of engineering but also the worlds of healthcare and even management economics. The development of CED within healthcare facilities in a structured manner is of recent occurrence and it has been expanding significantly in recent years. However, it is still present a significant variety in methods and in application's areas. For this reason, in order to facilitate greater contextualization with respect to the digitization pathway that will be illustrated, the reference context and the activities in charge of Clinical Engineering Department of the “Spedali Civili Hospital in Brescia” will be described.

The Complex Structure (CS) Clinical Engineering of the “Spedali Civili Hospital in Brescia” is responsible of the management—at company level—of medical and technical-economic equipment throughout their entire life cycle (technical specifications, evaluations, purchase, management, maintenance, end-of-life), of drawing up the investment plan, of the implementation of new projects, of innovative technologies in the biomedical field and of supporting the management for all strategic activities and issues related to medical technologies, medical devices and technical-economic equipment of all the facilities under its jurisdiction. The primary objective is to ensure the safe, appropriate, and efficient use of biomedical technologies and to draw up programs aimed at their best possible management. The main activities consist of:

- Planning purchasing of technological equipment, in cooperation with the company's biomedical technology committee.
- Definition of the technical specifications of biomedical and technical-economic technologies, verifying with departmental referents the specific needs to be met.
- Direct (preventive and corrective) maintenance or maintenance control for the technologies provided.
- Installation, testing, inventory of new equipment.
- Management of work related to the installation of technologies, in collaboration with the technical department of the company; management and distribution of medical, technical, and cryogenic gases and their cylinders in support of the company pharmacy department; management of medical IT in cooperation with the company information systems department (ICT).
- Development of HTA studies, in connection with regional organization.
- Studies on the implementation of innovative technologies and their Operational Management in current healthcare facilities and in the structures/hospitals of the future.

The CS Clinical Engineering operates according to a quality management system certified according to ISO 9001:2015. Furthermore, it is part of the “Facility Management and Safety” (FMS) team, which follows accreditation according to Joint Commission International standards.

Lastly it should be pointed out that, in the manner and under the terms of an existing contract, the CS cooperates with an external company to perform maintenance activities and electrical safety checks inside the hospital.

### 1.2. The fundamental role of an effective management program

Medical equipment is one of the key components contributing to the effectiveness of health services (17). The procedures involved in health services, ranging from diagnosis to treatment, rehabilitation to screening, prevention to monitoring, depend on the efficiency of medical equipment (18). Therefore, the provision of health services is almost impossible without proper maintenance of medical equipment (19). In addition, devices must be monitored to maintain performance in terms of calibration, maintenance, restoration, training, and decommissioning (20). As mentioned above, clinical engineers in a healthcare facility are responsible for regulating and introducing an effective management program for the reliability and safety of medical equipment (21). Therefore, maintenance management of medical equipment is critical to ensure that medical equipment operates according to the manufacturer's specifications and ensures the safety of patients and users (22). Proper implementation of maintenance can prevent failures or breakdowns that affect healthcare operations and can cause serious injuries to patients. Kutor et al. (23) reported that equipment failures are commonly due to inadequate transportation and storage, preliminary failures, mismanagement, lack of maintenance, environmental stress, random failures, improper repair methods, and wear and tear failures. Also important is the fact that 50-80% of equipment failures are due to poor maintenance and lack of highly trained technicians. In addition, the four main causes of these failures are: avoidable incidence, insufficient technical personnel, lack of data, and lack of predictive maintenance. Therefore, the maintenance and management of medical equipment can be progressively improved by identifying the influencing factors. Bahreini et al. (24) stated that unprofessional execution of maintenance affects health care performance, safety, and overall expenses of health care institutions, while Wu et al. (25) showed that effective maintenance management can reduce operating costs by more than one million dollars and improve equipment availability. Key factors in these rates are the increasing motivation for preventive maintenance, demand for equipment, implementation of advanced financing mechanisms, purchase of refurbished equipment, and implementation of a strict regulatory framework. These data show that the substantial budget for the purchase and maintenance of medical equipment is imposed to provide effective health services. In conclusion, it can be said that the current availability of medical equipment data in terms of equipment details, purchasing information, operational performance, and maintenance activities is critical to improving equipment life cycle management. However, the appropriate technique is critical to manage big data that provide meaningful indicators for maintenance management planning (26).

As cited by Zamzam et al. (27) four gaps have been highlighted by literature review which are:

- Lack of studies concentrated on comprehensive maintenance management, including preventive maintenance, corrective maintenance, and replacement program (28–30).
- Inconsistency of mathematical approaches that require manual intervention in identifying the criteria weightages in reliability assessment.
- Inefficiency of the previous predictive models, which can be applied to the several types of medical equipment (31–34).
- None of the studies combines assessment and predictive models using the same unlabeled dataset of medical equipment.

In the light of these evidence and in spite of the evident correlation with the provision of better healthcare services, it is noticeable that this area of study is still underdeveloped (35–38).

It is therefore clear that the proper management and maintenance of biomedical equipment is closely related to the delivery of more efficient healthcare services but also to a better utilization of company resources (39). So, in order to manage new technologies efficiently the numerous technical, economic and usability factors associated with clinical equipment must be taken into account. Consequently, it is of utmost importance that technical decision makers—this means Clinical Engineers—acquire the appropriate method and information for equipment planning and acquisition (40).

### 1.3. Purpose of the article

With all these considerations kept in mind, the CS Clinical Engineering of the “Spedali Civili Hospital in Brescia” decided to take up the challenge and to design a path that would lead to the development of a software for the management of biomedical technologies within its competence inside the hospital. The ultimate aim of this path stems from the need of Clinical Engineering Department to have up-to-date, realistic, and systematic control of all biomedical technologies present in the company. This path stems from the necessity to respond to the needs illustrated in the previous paragraphs and it finds in the digitization of processes an adequate response to these demands. The aim is to share the followed path and, on the basis of this, to develop a series of reasoning and considerations on how a good digitization process could take place and be successful. In this sense, there is evident transversality with recently discussed topics such as telemedicine and proximity medicine which, although articulated on the basis of different needs, find points of convergence with this digitalization process. With respect to the latter consideration, however, it should be considered that telemedicine involves medical practice and information and communications technology. It has been proven to be very effective for remote health care, especially in areas with poor provision of health facilities. However, implementation of these technologies is often hampered by various issues. In particular technical, ethical, medico-legal, and legal aspects must be considered (41–43). The project illustrated in this article considers these issues but it deals with minor limitations due to the fact that it does not handle with sensitive patient data but only with technical and managerial specifications of the biomedical equipment used to produce them. In light of this, anyway, the software platform must still comply with the provisions of the General Data Protection Regulation (GDPR) No. 2016/679.

This case study is articulated on the basis of a very complex logistical structure, and this can therefore make it scalable to other realities of equal or lesser complexity. In fact, the “Spedali Civili Hospital in Brescia” is not just one of the most important corporate realities in the city, but it is also the largest hospital in Lombardy and one of the largest in Italy. In addition to the central referral center, it has three other hospital presidia and several outpatient clinics and afferent facilities in the territory. The “Spedali Civili Hospital in Brescia” has been recognized as the second-best hospital in Italy in 2013 and it is characterized by the presence of a machine park of about 30.000 units that, despite its high volume, requires operational management and organized monitoring.

## 2. Materials and methods

**Figure 1:**
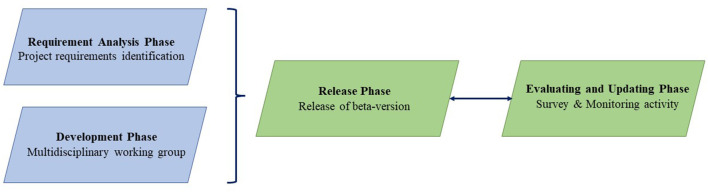
The four distinct phases followed in order to develop the software.

Appropriate and efficient use of this integrated software is inevitably related to the proper functionality and differentiation of each specific function and to the subcomponents' synchronized work. System development has followed the well-established phases (Figure 1):

### 2.1. Requirement analysis phase

The identification of both the currently existing management criticalities and the consequent project requirements, has represented only the first step of this path. The main objective of such an integrated software system is to assist the Clinical Engineering in performing tasks concerning safety, effectiveness, and efficiency in use of medical equipment. Requirement analysis indicated that the system should provide the following features:

- Management of files for medical devices, manufacturers, and suppliers.
- Follow up of purchasing procedures, from the request of the departments through acceptance tests of the devices.
- Implementation and management of quality and safety protocols and procedures—including the necessary documentation and data—presented in an appropriate and comprehensible format.
- Scheduling of all routine procedures such as acceptance testing, preventive maintenance, and quality and safety inspections.
- Follow up of corrective maintenance tasks.
- Monitoring of the overall performance of the department, using quality and cost indicators.
- Easy access to and exchange of vigilance-related information.
- Data analysis and report generation, either predefined or customized by the users.

Also, the main general technical specifications of the architecture related to hardware and software structure are explained next. Their identification, which took place through brainstorming techniques, is based on the technical, theoretical, and logistical needs of CS Clinical Engineering:

- Ability to use the core functionality of the platform without the need for installation/setup on end-users' computers. For this reason, it is preferable for the application to have a “web application” type user interface so that it can be enjoyed through common web browsers. Exceptions are allowed related to specific features that have the unavoidable need for interfacing with dedicated hardware and/or that cannot be achieved *via* web browsers. A simplified software upgrade process that does not require desktop client installation and upgrades is also required.
- User authentication: the software platform must have functionality for user authentication and secure access, as well as a log-in mechanism for user authentications.
- User profiling: the software platform must have dedicated functionality for user management and user profiling. It must be possible for System Administrators users to manage through an interface the configuration of users, their profiling, and the definition of user roles.
- User management and user licensing: the number of users that can be configured within the software platform must be unlimited, and it must be possible to provide for a simultaneity of at least 150 users.
- Handheld software management: for easier direct on-site management, handhelds or mobile devices must be provided that allow for the reading of any barcode, QR or RFID codes in order to be able to make on-the-spot changes to the last detection of the equipment, as well as to be able to use all software functions in synchronous and asynchronous modes. Device synchronizations must be able to be simultaneous, scheduled (at least once a day) and with the possibility of performing them on demand.
- Integration with monitoring portals of external companies: the possibility is required, for high technologies and where available, to link directly to the monitoring portal link of the Company responsible for the maintenance contract in place.
- Possibility of massive uploading of data and/or documents related to equipment management by the supplier.
- The software platform must comply with the provisions of the General Data Protection Regulation (GDPR) No. 2016/679.
- Integrated authentication with enterprise Active Directory on Premise. Two-way integration with Archiflow, Siaweb, EUSIS/DigitGO, Coswin software and enterprise folders. Data interchange between systems must allow for “one-time” import of data sets, two-way live integration to obtain data from the above systems or to be able to enter/update information entered on the new software platform, two-way live integration for document retrieval archiving with Archiflow software.
- User friendly and customizable interface.
- Cloud hosting that ensures data security and scalability.

The above specifications as well as the contemporary trends in the clinical engineering sector worldwide, were taken into consideration throughout the Development Phase of the system.

### 2.2. Development phase

Firstly, a technological partner—for the development of Information Technology (IT) section related to the software—has been identified through an open procedure. Subsequently, it was deemed appropriate to form a multidisciplinary group composed by the Clinical Engineering staff and ICT department—for IT security issues—of the “Spedali Civili Hospital in Brescia” and a research university group from the Department of Electronics, Information and Bioengineering (DEIB) of “Polytechnic of Milan”. All of them in collaboration with the technological partner identified in “E.L.L.F. S.r.l.”; a series of meetings were then held for one year to design and develop the digital system. Steps of this process, specifically, were the following: *definition of software technical specifications, flow analysis and optimization*, and *software design*. Meetings were characterized by a detailed analysis of Clinical Engineering's processes: management of the registry of equipment present in the facility, orders and contracts, pre-testing phase and subsequent testing of new equipment that has been taken over, management during the entire life cycle and end-of-life. The flows, in addition to being studied in the form in which they are currently carried out, have been optimized in order to lend themselves better to the digitization process. Finally, the work was focused on the design of the portal. Starting with an analysis of the graphical user interface of the management software previously in use and how the processes were previously managed, it was discussed how the same—after being revised and optimized—could be made available in digital form in a way that was as intuitive and user-friendly as possible.

Various techniques and methodologies were used during the meetings to carry out the characteristics and the requirements that the software had to satisfy. Solutions were discussed and selected through common brainstorming techniques. Regarding the process analysis, Clinical Engineering reviewed in detail all phases and steps of the current internal procedures. Subsequently, the data involved within a given process and their flow within the processes themselves were highlighted: data used as input and the corresponding data produced as output were identified for each process. In order to conduct these process analyses, *User Stories* and *Use Cases Diagrams* were developed. The latter made it possible to capture the functional requirements that the software must fulfill within a given process.

### 2.3. Release phase

Release Phase consisted of two different stages. Initially, the release of a beta-version of the software—including the main functionalities—was launched. The beta-version was for the exclusive use of Clinical Engineers and Clinical Engineering Technicians (CET) and the purpose of this preliminary release was exclusively to test the correct implementation of the first functionalities and to verify that they met the previously identified needs. Subsequently the definitive version of the software, called *Asset Manager*, was released for the use of all end-users.

In view of the remarkable structural and logistical complexity of the “Spedali Civili Hospital in Brescia”, the software Release Phase followed a very precise logic. The release of the software took place at first at the territorial hospital presidia and only later at the referral center. This choice is due to the fact that most of the overall volume of biomedical equipment and coordination and management flows of Clinical Engineering are concentrated at the central presidium. For this reason, it was deemed appropriate to release the new software in a progressive manner and according to an increasing gradient of complexity. This approach is indicated in bibliography as a gold standard for the delivery and implementation of new services.

### 2.4. Evaluating and updating phase

Evaluation, an integral part of the system development to ensure system functionality, was performed in three distinct phases which included testing, verification and validation. Testing procedures were performed by internal evaluators of the technological partner as well as software professionals with significant knowledge related to the system structure. Testing goal was to determine the proper functioning of the system, to monitor problems related to database management, and to identify weak points in the software packages. The integrated system also was distributed for verification to a set of end-users; here it should be emphasized that before the final release of *Asset Manager* platform, functional (i.e., simulation of activities and procedures) tests were conducted to simulate all functionalities and their enforceability by the entire CS.

End-users were instructed through appropriate training programs. The objective of gathering their feedback was achieved through the development and distribution of a survey. The macro-areas of the survey are those relating to the management section of the software, and for this reason the questionnaire was only addressed to the Clinical Engineering personnel, even though they are not the only users of the software.

Bottom line, the path could not disregard from a constant monitoring activity of the progressive implementation status of the software in order to supervise the functional integration of the new system into the operational reality.

## 3. Results

### 3.1. Clinical engineering management software (SIC Brescia)

First of all, the name that has been chosen for this new management software is “*SIC Brescia*”.

Discussion about needs and requirements of Clinical Engineering led to the identification of the desired characteristics which were then summarized in a list of technical specification required for the software. Cooperation between the various figures involved in the multidisciplinary group led to the development of an innovative software realized through the application of the latest available web technologies, characterized by user friendly and customizable interface, single page application usable through all modern browsers, cloud hosting for data security and scalability, integrated authentication with corporate Active Directory on Premise and simplified software update process that does not require installation and upgrade of desktop clients.

The input provided by the multidisciplinary group was crucial in highlighting, by virtue of their areas of expertise, a number of needs and design specifications to be taken into close consideration during the software *Development Phase*. It is emphasized that multidisciplinary can only be seen as an added value in the implementation of complex projects, such as those involving health care facilities nowadays. In particular, the technical-operational section of CS Clinical Engineering as well as the medical/clinical personnel that will interface with the use of the software consider the following aspects to be fundamental: simplicity in searching for a specific piece of equipment through the use of search filters, immediacy in finding information related to it, detail in compiling the technical-file of each device, facilitation of workflows, ease in attaching and subsequently consulting documents related to a piece of equipment, intuitiveness of software's graphical interface, and efficiency and comprehensiveness of processes. The management component of CS Clinical Engineering needs the possibility to retrieve overall and final data about all the equipment under its purview with a specific degree of detail about maintenance operations and equipment testing processes. The ICT department mediated the integration of the new software with the company's IT architecture, ensuring the functionality of the systems already in place and the security of the data processed. The technology partner mediated all the requirements presented to make possible their effective fulfillment in the software implementation. Finally, the research group was involved, and it is actually involved in the development of indicators for the creation of new OM tools and indicators.

**Figure 2:**
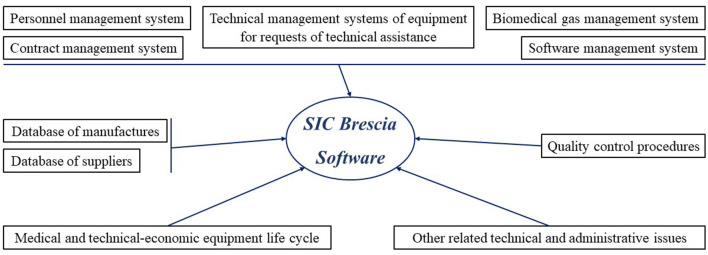
All aspects related with Clinical Engineering Management that has been implemented in software's architecture.

From a technical point of view this system was designed to manage all aspects related with Clinical Engineering technical management, as summarize in Figure 2, inclusive of:

1. Contract management system, both for the acquisition of new equipment and maintenance of the same.
2. Personnel management system.
3. Medical and technical-economic equipment life cycle from acquisition to dismission and this includes the following aspects: inventory, classification, technical specification, warranty period follow-up, equipment service support including preventive and corrective maintenance, analysis of performance, equipment dismission and out of service, electrical safety check, testing of an equipment.
4. Software management system.
5. Biomedical gas management system.
6. Database of suppliers and related contacts.
7. Database of manufactures and related contacts.
8. Technical management systems of equipment for requests of technical assistance.
9. Quality control procedures.
10. Other related technical and administrative issues.

The system also includes an efficient reporting scheme that can produce immediate reports on all aspects concerning medical equipment such as list of equipment in any location, equipment list of certain type or manufacturer, list of equipment failures, all information (severity, duration, current state etc.,) related to technical intervention and preventive and corrective maintenance reports. From a systemic point of view the release of beta-version allowed an initial analysis of preliminary evidence and it revealed how the new digitization path led to:

- *Speeding up procedural flows* by computerizing the company's administrative and technical processes currently managed entirely on paper.
- *Development of operational management activity* by monitoring the effectiveness and efficiency indicators of biomedical technologies in charge through the creation of a *Management Dashboard* within the software.
- *Development of indicators to support Health Technology Assessment (HTA)* by monitoring maintenance activities performed and the degree of equipment utilization.
- *Development of a prioritization algorithm* for the optimized handling of requests for assistance made by hospital operating units.
- *Increased traceability of* actions taken through the completion by suppliers of a dedicated form to collect input data of all tested equipment.
- *Integration of the system* with software already in use and with company IT tools in accordance with the ICT department.

### 3.2. Survey's results

Results of the survey revealed that, with regard to the comparison with the previously used—not company property—management software, all respondents agreed that *SIC Brescia* was more intuitive and efficient. Furthermore, with regard to the overall assessment, all users felt that the new software was intuitive, user-friendly, and easily comprehensive Also, it has been highlighted that a large number of steps were not required to perform the desired tasks. Finally, on the basis of a scale of one to five, all users were more than satisfied with the overall use of the new digital software.

## 4. Discussion

### 4.1. Contest of reference analysis

This article deals with all distinct phases of design and implementation of a fully automated clinical engineering technical and operational management software called “*SIC Brescia*” at “Spedali Civili Hospital in Brescia”. The conception of an innovative digitization process stems from the need to coordinate and manage the number of new technologies that has increased exponentially in recent years. Digital innovation is nowadays affecting many aspects of healthcare processes: from the management of appointments to the administration of pharmacological therapies, from the organization of territorial care and the new way of delivering services remotely to the management of biomedical technologies, with a consequent impact also on the various professions that are involved. Modern technologies require more dedicated professionals, not only health professionals but also technical professionals, who must ensure the safe delivery of services (17–20, 44, 45). Clinical engineers find themselves among the main players in this transformation, and it can be stated that all of this necessarily implies the adoption of new management logics that allow a true integration of the most diversified needs connected to the broader and more complex healthcare context (46). In fact, a real transformation in digital terms requires more than a simple change in the technological, structural and organizational assets that its implementation imposes, thus producing a new management that contemplates new aspects or new opportunities. A context that wants to evolve and aims at quality and performance efficiency must dispose of structured and multi-parametric information; this implies facing new challenges such as the establishment of new information flows that require a logic of compatibility, interoperability and integration between technologies and the rest of the corporate and regional information systems. The collection of structured data can also be used to build dashboards with summary data and KPIs. Furthermore, it enables the optimization of resources according to economic (management costs) and organizational (resources, spaces) requirements, quality, regulatory and security reference standards. Hence the need for a systemic vision and dialogue between the various professionals: in order for the digitization process to be conducted effectively, internal processes and procedures must be rethought and recoded to ensure the true applicability and application of the new digital technologies. In this context, the concept that led to the realization of the new management software “*SIC Brescia*” was developed. The authors emphasized that they could state with significant certainty that the general technical specifications in the *Requirement Analysis Phase* section are predominantly scalable and contextualizable with so many other CED realities. The entire process of realizing the platform was not born with the exclusive intention of digitalizing the data and information relating to the activities carried out by Clinical Engineering. On the contrary, it should be taken as an opportunity to rethink processes by making them simpler and more streamlined. It is therefore clear that the aim must not only be to create a “paper-less” system, but to obtain software that integrates itself into the operational and technical reality, facilitating the completion of activities and the carrying out of processes. Only in this way technological innovation, and specifically this digitization path will be able to realize the much desired “added value” to be gained from innovative processes. As a corollary to this reflection, it is especially important not to lose sight of the principle by which technological innovation must be flexible and capable of modulating itself to the needs of the realities in which it is structured, and not vice-versa. Only in this way a constructive integration capable of creating value, not only for the company but also for the citizen, could be finally observed.

### 4.2. “Value added” theme & change management process

**Figure 3:**
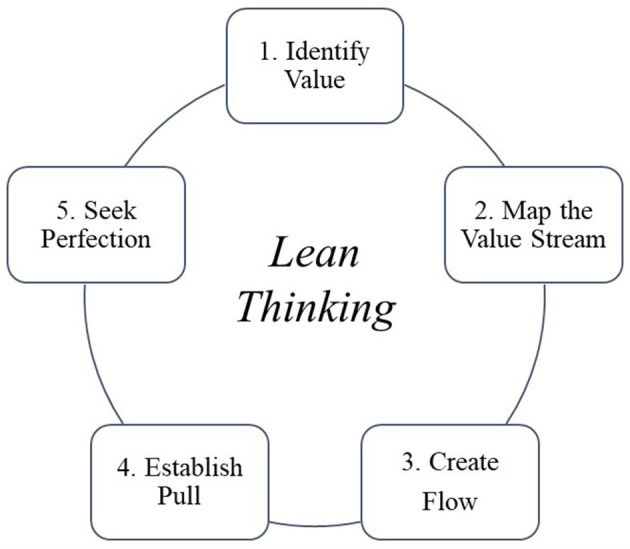
The five steps of lean thinking implementation.

Another important aspect to be considered when major changes are introduced into processes—all the more so in the healthcare context—is a careful study of the processes being innovated. Lean Thinking [Figure 3, (47–50)] provides a number of tools, including the fundamental “*value stream map*” (51). It starts with an analytical “*as is*” snapshot of the current situation, followed by a prospective “*to be*” view of how the process will change in relation to the findings and the technologies introduced. First of all, the concept of “*value*” must be defined. Value for a process of this type can only be the response to adequate management of equipment and operational processes. In order to do this, many activities have been carried out: of these, all those activities in an operational process that are actively carried out on the life cycle of a technology are considered “*value added*”. Surrounding these activities—from which the entire organization benefits—there are many others. Some of which are necessary for the processes and acts of healthcare to be carried out, the so-called “*business value added*,” and others which could and should be done without, the so-called “*non-value added*”. The objective of the *Requirement Analysis Phase* was precisely a lean review of the operational processes of biomedical technology management, which aimed to eliminate non-value-added activities and increase and optimize value added ones. The correct description in a flow chart (*value stream map*) (52), according to the concepts just outlined, of all the activities, human and instrumental resources employed, and the time required to provide the services themselves, has provided a snapshot of the current state of the processes and the possibilities to improve them. Given this premise it seems clear that in general the best innovative digital technologies are those that, inserted in a process, in addition to improving the qualitative performance of health-technological management, are aimed at reducing the previously cited non-value-added activities. In all this context, what is known as “change management” should by no means be underestimated. The more innovative digital processes are, the more they change the way in which they are delivered by the practitioner and also the way in which the practitioner can use them. The risk is to create feelings of discomfort that—if the change is not properly managed—may lead to their rejection. Reference is also made to those technologies that are currently defined as “disruptive innovation” due to their strong innovative nature and for which process mining and change management activities are necessary. Change management should help professionals to achieve optimal everyday use of new methodologies by supporting each one of them, in an almost personalized way, during each phase of the new process. Change management is a process structured in precise steps. These steps have been followed in the realization of this project and they have been summarized in the following cornerstones: the change that new technologies introduce must be part of a clear project design (*vision*), be able to count on adequate professional competences (*skills*), with adequate incentives (*incentives*), the right resources (*resources*) and a clear action plan (*clear action plan*). The lack of only one of these elements, or the poor definition and/or incomplete implementation of one of the steps described above will certainly lead to the failure and breakdown of the innovation that was intended to be introduced. Change management helps professionals to value change, to accept it, and to correctly identify areas of improvement for their business (53). This procedural approach can be declined to a broader and more generalized character with respect to contemporary reality: we are faced with increasingly innovative technological development and there is an urgent need to discern which innovations are most useful and applicable. We need to be able to discern among all the technologies which really allow us to change healthcare processes by making them leaner, more efficient, and more useful to the patient which is the end user of the process but who should actually be considered the trigger for any improvement action. There is therefore a responsibility to optimize the resources made available to us and allocate them correctly in the knowledge that they will never be enough and that it is therefore up to us to discern useful innovations from those without added value for the patient. This last consideration leads to the possibility of conducting a constructive reasoning about the contextualization of technological development in hospital context. If, on the one hand, the innovation brought about by the “*SIC Brescia*” software brings about a change in working mentality, revolutionizing the current parceled out working method, reorganizing and facilitating the ordinary activities of Clinical Engineering in a single digitized working solution, it is also true that the latter cannot fail to be accompanied by the development of an adequate digital culture in the system in which it is integrated. In this sense, the effort of company management, and even before that of institutions, in training and orientation toward adequate digital training plays a fundamental role. It follows therefore that continuous training and skills development—both technical and digital—for all the professionals working in the system are essential, in order to ensure full compliance and real implementation of new processes under way for the renewal of the care settings of the “Spedali Civili Hospital in Brescia”. Lastly, this discussion cannot end without considering the delicate issue of technology acceptance: a topic that is neither trivial nor inessential. In fact, the quality of the final use of the software passes through the mediation of the technology in the relationship with the user, not forgetting that the motivation of the need or usefulness of the technology passes through concepts such as usability, perception of benefit and comprehensibility. It is precisely for this reason that the multi-disciplinary character held during the realization and design of the software and the drafting of a questionnaire to assess the usability of the software itself were important aspects to take into consideration concerning to the topic of technological acceptance.

### 4.3. Transversality with digital healthcare

The potentialities of this digitization pathway also find transversal points of contact with other tremendously contemporary themes inherent in the innovative digital processes currently taking place in the healthcare sector. Although the peculiarities associated with other themes have a different functional character and application context, the considerations mentioned above also find a contextualization in them. In particular, a direct connection can thus be drawn with the topic of telemedicine. Here too, only a careful and objective assessment of the processes and the benefits that the technologies bring to them and to the patients can provide the measure—both in terms of the priority of intervention and in terms of the need—to intervene and improve the healthcare process. In the absence of such careful study, the risk is that of following the trends of the moment and under- or over-estimating innovative technologies compared to the value they would bring to healthcare processes. In this context, in addition to complying with the current rules and regulations governing their marketing and use, the new challenge for the Clinical Engineer is therefore how to map and manage remotely distributed medical equipment and devices, even more so in a home-based regime (in a logic of proximity medicine), throughout their entire life cycle considering all the possible needs that may arise, ranging from simple periodic maintenance to extraordinary maintenance to technological and/or software upgrades, to the renewal of any necessary consumables and the safe use of the medical device according to its specific destination. A new range of action and supervision, therefore, asserts itself in this scenario for the Clinical Engineer who advances from a hospital-centric logic to a digitized territorial logic.

The development of the new “*SIC Brescia*” software was created to meet these requirements as well. It also considered the current implementation of the new Regulation on Medical Devices (MDR)—Regulation (EU) No. 2017/745—thanks to the presence of a section for reporting the UDI (Unique Device Identification) code in the master data section of each piece of new equipment. This made it possible to comply with the strict traceability requirements in accordance with the new regulation.

### 4.4. Toward a new concept of Clinical Risk Management

Effective operational management cannot disregard, among other aspects, from the concept of Clinical Risk Management (54, 55), which aims to improve the quality and safe delivery of healthcare services through procedures designed to identify and prevent circumstances that could expose a patient to the risk of an adverse event. Throughout its history, clinical engineering was focused on medical devices and how they are used in the healthcare environment. However, during time, clinical engineers have become deeply involved in quality improvement and risk management activity. The healthcare technology management aims to optimize the acquisition and utilization of medical technology to achieve maximum beneficial impact on health outcomes (56, 57). In particular, proper maintenance implementation can prevent failure or breakdown that affects the healthcare operations and may cause severe injury to the patients (44). Development of a new software is fundamental in order to acquire a list of new information and to organize these ones with a set of new KPIs (58, 59). The development of these indexes allows an evolution of the Clinical Risk Management concept that tends toward an integrated model with increasingly organized and complete data. The digitalization process allows the systemic organization of a series of information that, in an organization without this type of software, could not be developed. Hence, we are moving from a “static” Clinical Risk Management model to an increasingly “dynamic” model that finds its consistency in a series of progressively more articulated data. However, it must be remembered that a large amount of data needs to be organized in order to produce valid information. Consequently, it is of absolute importance that Clinical Engineers acquire the appropriate methods and information regarding equipment planning, acquisition, and evaluation. According with the recent literature some examples of KPIs that could be implemented in next future are: Global Failure Rate (GFR), Age Failure Rate (AFR) and Acquisition Trend (AT). The GFR is calculated using the total number of failures by the total number of completed repair work-calls divided by the total number of devices (45, 59). This measures the reliability of medical equipment, a fundamental aspect in guaranteeing hospital medical services (33, 60). The AFR represents the total number of failures divided by the total number of devices according to the number of years used and provides us with more information than the GFR as it takes into consideration user experience and learning ease. The AT of medical devices provides further information for long term equipment replacement by providing a periodical purchasing trend which can be applied toward economic resource planning. In addition, the presence of a management dashboard makes it possible to monitor the progress of ordinary maintenance and the integration of this data with the aforementioned KPIs will make it possible to evaluate optimization of the maintenance process (12–15, 17). The maintenance process and program can be improved through the development of models that test changes in the periodicity and in the methodologies of maintenance activities with the final aim of increase the lifecycle length and management of biomedical technologies (19–24). So, this new methodology will provide some useful information for maintenance and technology replacement phases and KPIs for decision-makers in technical analysis within technology management. Further improvements are connected to technical dashboard development for sustainable technology management by including usability and economic indicators. This would assist decision makers in technology replacement and management phases, allowing for an efficient view of technological information in health structures and for a better realization of the concept of Clinical Risk Management.

## 5. Conclusions

The contribution of the present paper is to illustrate the development of a complex implementation model for the digitization of processes, information relating to biomedical technologies and their management throughout the entire life cycle. This process was made possible by the implementation of management software and was carried out by a multidisciplinary working group at the CS Clinical Engineering of the “Spedali Civili Hospital in Brescia”. The purpose of sharing this path is to highlight the methodologies followed for its realization, the results obtained and possible future developments. This may enable other realities in the healthcare context to undertake the same type of pathway inspired by an accomplished model. The theme of “added value” associated with digitization processes can thus be realized and find final fulfillment. In fact, the realization of the model, from conceptual idea to the practical implementation, has been designed with the terminal aim of making it scalable and extendable to other hospital facilities. Scalability will be the subject of future deeper analysis, after the steady-state phase of the software will be fully operational and fully integrated at the “Spedali Civili Hospital in Brescia”. Furthermore, the implementation and data collection related to the previously proposed KPIs, as well as the consequent development of new operational management models for biomedical technologies and maintenance processes will be studied. In this way, the Clinical Risk Management concept will also be able to evolve into a more controlled, safe, and efficient system for the patient. Finally, the impact of this process finds fulfillment at the systemic level by promoting the transition from a working method historically set up with the “silo concept” (a vertical approach separated by competence in which people tend to think independently) toward a new more linear and integrated logic between the various professionals involved in the operational processes. The latter finds realization in the optimization of the overall functioning within the entire organization and in the common interest.

## Data availability statement

The original contributions presented in the study are included in the article/supplementary material, further inquiries can be directed to the corresponding author.

## Author contributions

MV, GV, MC, CD, LI, and PP participated in research design and in developing the entire project. MV wrote the manuscript. GV, MC, CD, LI, and PP participated in process analysis. VC and GV participated in the improving and revising of the paper. GV provided substantial advice in designing the study and VC assisting in the division of labor, writing, and revising of the paper. All authors listed have made a substantial, direct, and intellectual contribution to the work and approved it for publication.
